# Modulation of feeding behavior and metabolism by dynorphin

**DOI:** 10.1038/s41598-020-60518-0

**Published:** 2020-03-02

**Authors:** Aishwarya Ghule, Ildiko Rácz, Andras Bilkei-Gorzo, Este Leidmaa, Meike Sieburg, Andreas Zimmer

**Affiliations:** 1 Institute of Molecular Psychiatry, University of Bonn, Medical Faculty, Venusberg-Campus 1, 53127 Bonn, Germany; 2 Present Address: Department of Neurodegenerative Diseases & Geriatric Psychiatry University of Bonn, Medical Faculty, Venusberg-Campus 1, 53127 Bonn, Germany; 3 Present Address: Aarhus University, Department of Biomedicine/DANDRITE Capogna group, Ole Worms Alé 6, 8000 Aarhus C, Denmark

**Keywords:** Feeding behaviour, Molecular medicine

## Abstract

The neuronal regulation of metabolic and behavioral responses to different diets and feeding regimens is an important research area. Herein, we investigated if the opioid peptide dynorphin modulates feeding behavior and metabolism. Mice lacking dynorphin peptides (KO) were exposed to either a normal diet (ND) or a high-fat diet (HFD) for a period of 12 weeks. Additionally, mice had either time-restricted (TR) or *ad libitum* (AL) access to food. Body weight, food intake and blood glucose levels were monitored throughout the 12-week feeding schedule. Brain samples were analyzed by immunohistochemistry to detect changes in the expression levels of hypothalamic peptides. As expected, animals on HFD or having AL access to food gained more weight than mice on ND or having TR access. Unexpectedly, KO females on TR HFD as well as KO males on AL ND or AL HFD demonstrated a significantly increased body weight gain compared to the respective WT groups. The calorie intake differed only marginally between the genotypes: a significant difference was present in the female ND AL group, where dynorphin KO mice ate more than WT mice. Although female KO mice on a TR feeding regimen consumed a similar amount of food as WT controls, they displayed significantly higher levels of blood glucose. We observed significantly reduced levels of hypothalamic orexigenic peptides neuropeptide Y (NPY) and orexin-A in KO mice. This decrease became particularly pronounced in the HFD groups and under AL condition. The kappa opiod receptor (KOR) levels were higher after HFD compared to ND feeding in the ventral pallidum of WT mice. We hypothesize that HFD enhances dynorphin signaling in this hedonic center to maintain energy homeostasis, therefore KO mice have a more pronounced phenotype in the HFD condition due to the lack of it. Our data suggest that dynorphin modulates metabolic changes associated with TR feeding regimen and HFD consumption. We conclude that the lack of dynorphin causes uncoupling between energy intake and body weight gain in mice; KO mice maintained on HFD become overweight despite their normal food intake. Thus, using kappa opioid receptor agonists against obesity could be considered as a potential treatment strategy.

## Introduction

Obesity is a major contributing factor to many health problems in industrialized countries. This fact is widely known, but the number of overweight and obese people is nevertheless increasing. Although the driving causes are multi-factorial, the fundamental cause of obesity is an energy imbalance between calories consumed and calories expended. The free accessibility to a highly palatable and calorie-dense diet is a potential contributor to the current obesity epidemic. Studies on animal models have been crucial to elucidate the mechanisms of nutrient homeostasis and imbalance, mainly by using pharmacogenetic and behavioral interventions. In mice, it has been shown that *ad libitum* access to a high-fat diet (HFD) causes obesity, insulin resistance, hepatic steatosis, hypercholesterolemia, and dyslipidemia^1^.

The regulation of palatable food consumption is an important determinant of obesity. There is considerable amount of evidence that neuronal and cellular mechanisms essential for regulating feeding behavior are similar to those engaged by drugs of abuse. Consumption of palatable food activates dopamine-releasing neurons that project from the ventral tegmental area (VTA) to the nucleus accumbens (NAc), the brain reward system^2^. HFD influences dopaminergic signaling by increasing the D2 receptor activity and decreasing the dopamine transporter binding density^3^. Beside the dopaminergic signaling, endogenous opioids are also postulated to mediate several aspects of feeding behavior^4^. The opioid system does not only regulate the hedonic appetite for palatable food, but also the amount of calories consumed during different times of the day^5–7^. Although the opioid system has long been implicated in controlling hedonic and homeostatic feeding as well as regulating body weight and metabolism, the individual contribution of different opioid peptides in feeding behavior is unknown.

The present study focuses on one member of the endogenous opioid peptide family, dynorphin (Dyn), which preferentially binds to kappa-opioid receptors. It mediates the rewarding properties of drugs of abuse and modulates feeding behavior^8^. Dyn serves as a negative feedback mechanism in the mesolimbic dopaminergic reward circuit^9^. Dyn released from the nerve terminals of the NAc acts on kappa opioid receptors located on dopaminergic nerve terminals and cell bodies and reduces dopamine release in the NAc. Inhibition of Dyn/kappa opioid receptor signaling disrupts this feedback loop and modulates the rewarding effects^10^. We hypothesized therefore that Dyn differently influences homeostatic and hedonic feeding.

Another brain structure involved in the regulation of feeding behavior, satiety and energy homeostasis, in which Dyn has been localized, is the hypothalamus^8,11^. The hypothalamus, also known as the “centre of feeding and satiety”, plays a key role in the control of food intake by sensing metabolic signals from peripheral organs and modulating feeding behavior^12^. A major role in these neuronal circuitries has been assigned to neurons producing the orexigenic neuropeptides such as neuropeptide Y (NPY) and orexins like orexin-A^13,14^. These neurons modulate the homeostatic feeding control and reward-seeking behavior. NPY is primarily synthesized in the arcuate nucleus (ARC). Stimulation of the NPY-ergic ARC - paraventricular nucleus (PVN) pathway by fasting, energy loss (glucosuria) and exercise is followed by increased appetite and food intake. As a result, parasympathetic activity is increased, sympathetic activity and energy expenditure are decreased^15^. Previous studies have shown an enhanced NPY expression in the dorsomedial hypothalamus in rodent models of obesity after chronic food restriction^16^. Furthermore, food deprivation results in a significant increase of NPY mRNA, which is co-localized with pro-Dyn in the hypothalamus^17^. Interestingly, in dynorphin-deficient mice NPY expression is downregulated^18^.

Orexins/hypocretins are exclusively produced in the lateral hypothalamus (LH) and project to the ARC among other areas^19,20^. They regulate appetite along with arousal and wakefulness. Orexin neurons are regulated by peripheral metabolic cues, including ghrelin, leptin, and glucose concentration, linking energy homeostasis to arousal state. Intracerebroventricular injections of orexins in rodents induce feeding behavior during the light period^14,21,22^, whereas orexin neuron-ablated mice show hypophagia and late-onset obesity^23^. A blockade of orexin signaling with the orexin 1 receptor antagonist SB-334867 reduces acute HFD consumption, thus acute HFD consumption requires orexin signaling^24^. Muschamp *et al*. showed that orexin facilitates cocaine reward in the ventral tegmental area (VTA) in mice during self-administration, by attenuating the anti-reward effects Dyn^25^.

The aim of the present study is to elucidate the role of Dyn in modulation of metabolic changes associated with HFD consumption using *ad libitum* (AL) and time-restricted (TR) feeding regimens in constitutive Dyn-deficient and wild-type animals.

## Experimental Procedures

### Animals

In this study, 61 constitutive prodynorphin gene null mutant (KO) and 75 wild-type (WT) littermates with a C57BL6/J genetic backgound were used^26^. KO mice were backcrossed for more than 10 generations to C57BL/6 J mice; therefore, they were congenic for this genetic background. The genotype of the animals was confirmed by polymerase chain reaction. Ten-to-twelve weeks old animals were group-housed and kept in a reverse light/dark cycle, (lights on: 19:00, lights off: 09:00 hours). Food pellets were provided either for a time-restricted (TR) period (10:00 to 18:00 hours) or *ad libitum* (AL), depending upon the experimental feeding group. The mice were housed at 21 ± 1 °C and 55 ± 10% relative humidity. Water was provided *ad libitum* in all cages throughout the experiment. All procedures were carried out in accordance with regulations for animal experimentation in Germany and were approved by the *Landesamt für Natur*, *Umwelt und Verbraucherschutz in Nordrhein-Westfalen*, Germany.

### Recording the body weight and food consumption

Mice were housed in groups of two to five animals per cage. Body weight and food consumption were monitored 2–3 times per week, over a 12-week period. Body weight data were analyzed separately by sex because of a difference in their weight baseline levels. The standard normal diet (ND) consisted of 1.28 kcal/g protein, 0.35 kcal/g fat, 2.26 kcal/g carbohydrates, with a caloric value of 3.89 kcal/g (Ssniff Spezialdiäten GmbH). The high-fat diet (HFD) consisted of soft food pellets (F3282, Bio-serv, Plexx, Europe) with 0.82 kcal/g protein, 3.24 kcal/g fat, and 1.43 kcal/g carbohydrates, with a caloric value of 5.49 kcal/g (Ssniff Spezialdiäten GmbH). Food pellets were weighed twice per week in the morning (for TR feeding groups additional recording in the evening) and the amount of food left in the cages was subtracted from the initially recorded amount. Pellet crumbles and spillage were also subtracted from the original value and recorded again to maintain maximum precision. Pellets smaller than approximately 5 mm were removed. To analyze the overall effect on body weight, we calculated the body weight change from the initial body weight measurements. Weekly body weight data is represented as average values per group. Food intake of mice was analyzed as the amount of food consumed per kg of body weight for each cage, with a caloric value of 5.49 kcal/g for HFD and 3.89 kcal/g for ND, respectively.

### Monitoring blood glucose levels

Food was removed from all cages overnight (16 hours) and on the following day blood glucose levels were measured. Using a fine syringe needle (23 G) the tail vein was pricked, a drop of blood was collected onto the glucose test strip and measured using portable glucose meter (Accu-Check Aviva, Roche Diagnostics GmbH). Blood glucose levels were recorded on weeks 4, 6, 8, 10, and 12.

### Immunohistochemistry

Animals were anaesthetized by injecting 100 µl/10 mg (v/w) narcotic solution intraperitonially and perfused using 4% PFA/PBS solution for fixation. Cryoprotection was achieved by using a 10% and consecutively a 20% sucrose solution overnight. Tissue samples were stored at −80 °C until further use. Brains were embedded in TissueTek^®^ and coronal slices of 16 µm thickness were cut using a cryostat (CM 3050 Leica) and mounted on glass slides. The slices were rinsed with PBS for 5 minutes and permeabilized with 0.5% Triton X-100 in PBS for 1 hour. The slices were rinsed twice for 10 minutes, with PBS and blocked for 1 hour with 1% BSA/PBS and 10% donkey serum in a humid chamber. Anti-NPY antibody (Abcam ab30914 1:1000), anti-prodynorphin (Gene Tex GTX10280 1:500), anti-kappa opioid receptor (KOR) (Santa Cruz sc-7494, 1:500) and anti-orexin-A antibody (Millipore PC362 1:500) were applied to slices, except for the negative controls, in which case 3% BSA/PBS was applied, and incubated at 4 °C overnight. The negative controls were used for the validation of the antibodies. The slices were incubated at 37 °C for 2 hours, washed with PBS and the respective secondary antibody (donkey-anti rabbit IgG coupled with Alexa Fluor® 488 (Invitrogen A21206) for NPY and orexin-A, DyLight549 conjugated donkey anti-guinea-pig IgG (Jackson Lab 81030) for prodynorphin (Pdyn) and Alexa Fluor® 647conjugated donkey anti-goat (Life Sciences A21447) for KOR) was applied in the dilution 1:1000. Slices were incubated for 1 hour at room temperature in dark conditions, washed with PBS for 4 times, 10 minutes each. Lastly, immunostained brain slices were embedded in Fluoromount-G^®^ media (Southern Biotechnology Associates, Inc.) and covered with glass coverslips.

### Image acquisition and analysis

Fluorescence images for NPY and orexin were obtained with Zeiss Axiovert 200 M fluorescent microscope using 10 x or 40 x objectives. For Pdyn and KOR, a threshold value was set for obtaining an optimal signal intensity and the area covered with positive signals was calculated using the ImageJ software (Version 2.0.0, NIH, USA). In case of NP Y staining, where the boundaries of the target area - nucleus arcuatus - could be clearly identified mean signal intensity, in case of orexin-A where the boundaries of the lateral hypothalamus are less defined the integrated signal density was calculated by the ImageJ software (Version 1.50i, NIH, USA). Regions of interest were defined using a free-hand selection, the ARC for quantification of NPY and lateral hypothalamus for orexin-A signal.

### Statistical analysis

Statistical analysis was performed using a STATISTICA software package (version 7.1 Statsoft, Inc., 2005; StatSoft GmbH). Three-way repeated measures ANOVA was used to analyze the average body weight gain and food consumption (genotype and feeding as main factors and time as within factor) followed by Šidák’s multiple comparison tests separately for diet and sex. To compare the weight changes during the experiment, one-way ANOVA with repeated measurement followed by Dunnetts’ test and linear regression analysis was applied separately to sexes, genotypes and diets. Two-way ANOVA for repeated measures followed by Šidák’s multiple comparison tests was used to detect significant changes in the blood glucose levels (genotype as main factor and time as within factor) separately for sex, diet and food. Hypothalamic peptide expressions were analyzed by two-way ANOVA followed by Šidák’s multiple comparison test. Correlation between the NPY, orexin staining intensities and body weight was analyzed using Pearsonś correlation. To compare expression changes in Pdyn and KOR in WT mice we used Studentś t-test. The data are presented as the mean ± standard error of the mean (SEM) for all measurements. All graphical data was prepared using GraphPad Prism software (version 6, La Jolla California USA). The datasets generated and/or analyzed during the current study are available from the corresponding author on request.

## Results

### Body weight gain

There was a significant difference in the body weight between sexes and genotypes at the start of the experiments. In general, females had generally a lower body weight than male mice (sex effect: F_(1,126)_ = 208.6, p < 0.001) and although genotype did not influence the body weights (genotype effect: F_(1,126)_ = 1.662, p = 0.199), the weight of KO females was higher than of WT females (genotype × sex interaction: F_(1,126)_ = 6.53, p = 0.0118) (Suppl. Fig. 1). Thus, instead of body weights we analyzed body weight gain values throughout the experiment.

**Figure 1:**
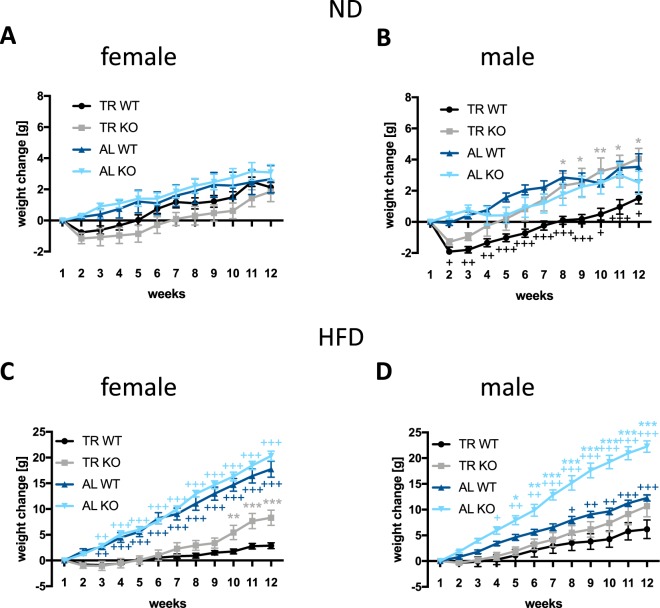
Body weight change of female and male mice receiving normal diet (ND) (**A**,**B)** or high fat (**C**,**D**) diet (HFD). Female and male mice, ND and HFD groups were analyzed separately. The animal numbers in the experimental groups were as followes: n = 7 in male ND AL KO, HFD TR WT and HFD AL KO; n = 8 in female ND TR WT, HFD TR KO, HFD AL WT, HFD AL KO and in males in the ND TR WT, ND TR KO, ND AL WT, HFD TR KO and HFD AL WT. N = 9 was used in female ND TR KO, ND AL WT and ND AL KO; n = 10 in female HFD TR WT. *p < 0.05; **p < 0.01; ***p < 0.001 difference according to Šidákś test between WT and KO in the TR (grey symbols) and AL (light blue) groups. ^+^p < 0.05; ^++^p < 0.01; ^+++^p < 0.001 difference between TR and AL groups according to Šidákś test in WT (black or dark blue) or KO (grey or light blue) mice. Symbols represent means, whiskers SEM.

Gain in body weight was analyzed separately for ND and HFD in female and male animals (Fig. 1 and Suppl. Tables 1–2). The weight of female mice kept on ND increased during the experiment (time: p < 0.001). This time-dependent change was not influenced by genotype, the way of food administration, nor did we find a genotype × administration × time interaction (Fig. 1A). Details of the three-way ANOVA analysis are shown in Suppl. Table 2. Detailed analysis of the data revealed that females in the AL groups increased their body weight continuously from the beginning of the experiment, whereas mice from the TR groups lost weight right after starting with the TR protocol in both genotypes (Fig. 1A). After this initial weight loss, from week 3 on, the weight gain was nearly linear and similar between the groups (Suppl. Table 1).

In male mice kept on ND, body weight change was influenced by the food administration method, because animals with AL access to food gained more weight than animals with TR access to food (administration x time interaction: p = 0.047). The difference between TR and AL groups was significant in WT but not in KO animals (genotype × administration × time interaction: p = 0.016). As observed in female mice, males also decreased their body weight after starting with the TR protocol, independent of genotype (significantly in week 2 for WT and in week 2 and 3 for KO, Fig. 1B), but from the third week on each group linearly increased their body weight. Unlike in females, the weight gain significantly differed between the groups of males; in KO TR mice, it was significantly higher than in any other group.

When the animals were maintained on HFD, females gained more weight under AL conditions (administration × time interaction: p < 0.001). The increase in body weight was significantly higher in KO than in WT mice in the TR group (genotype x administration x time interaction: p < 0.033). As shown at Fig. 1C, animals in the TR groups did not lose weight but just kept their initial weight for several weeks, so the weight gain became significant only in the 8^th^ week, whereas in AL mice it was already significant at the 3^rd^ week in both genotypes. The weight gain significantly differed between the groups; it was higher in AL compared to TR groups for both genotypes and the KO TR group gained weight quicker than WT TR (Fig. 1C, Suppl. Table 1).

Males in the HFD groups also gained more weight under AL conditions (administration x time interaction: p < 0.001). The increase in body weight was more pronounced in the AL group in KO than in WT mice (genotype x administration x time interaction p < 0.001) (Fig. 1D). Male mice in the TR groups, similar to females, started to gain body weight with a delay compared to the AL groups: The increase in body weight reached the level of significance in the 3^rd^ week in WT or KO AL groups, whereas only in the 6^th^ (KO) or 7^th^ week (WT) in the TR groups. Importantly, regression analysis revealed that KO males both in the AL and TR groups showed more intensive weight gain than WT males with the same feeding regime (Suppl. Table 1).

### Food intake

**Figure 2:**
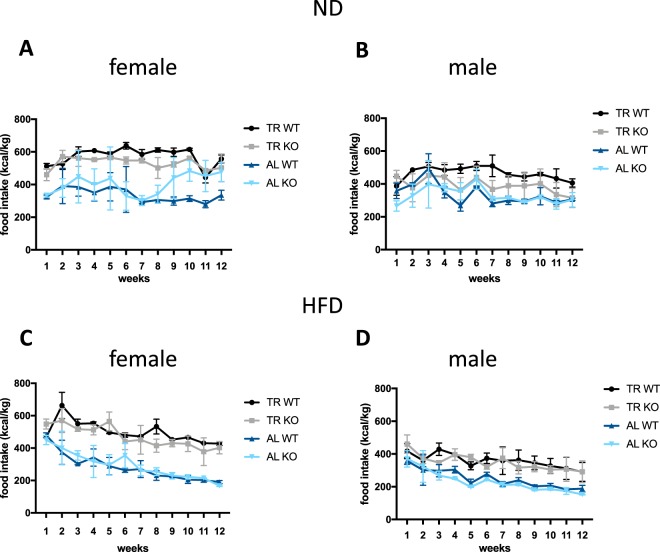
Food intake in WT and KO animals maintained on *ad libitum* (AL) or time restricted (TR) feeding schedules during 12-week feeding regimen. Food intake in female and male mice maintained on a ND are shown on (**A**,**B**), respectively, whereas, female and male mice maintained on a HFD schedule are shown on (**C**,**D**), respectively. Symbols represent means, whiskers SEM. *p < 0.05; **p < 0.01 according to Šidákś test compared to week 1 within the WT (black symbols) or KO (blue symbols) groups.

Food intake of female KO mice maintained on ND was increased compared to WT when the AL protocol was used, but was similar during TR regimen (genotype x administration interaction: p = 0.015) (Fig. 2A, Suppl. Table 3). Females in the TR group ate more than under AL condition (p < 0.001), whereas genotype, time or their interaction did not influence the food intake (Suppl. Table 3). In male animals on ND, the food consumption was influenced by time (time: p < 0.001) independently from the way of administration, genotype or their interaction (Fig. 2B).

In the HFD groups, we saw a very similar picture in both sexes: the amount of food consumed was significantly lower in the AL than in the TR groups (males: p = 0.003; females: p < 0.001) and food intake decreased during the experiment (males: p < 0.001; females: p < 0.001) (Fig. 2C,D). No other main effects or interactions were found (Suppl. Table 3).

### Blood glucose

#### Blood glucose: TR feeding schedule

**Figure 3:**
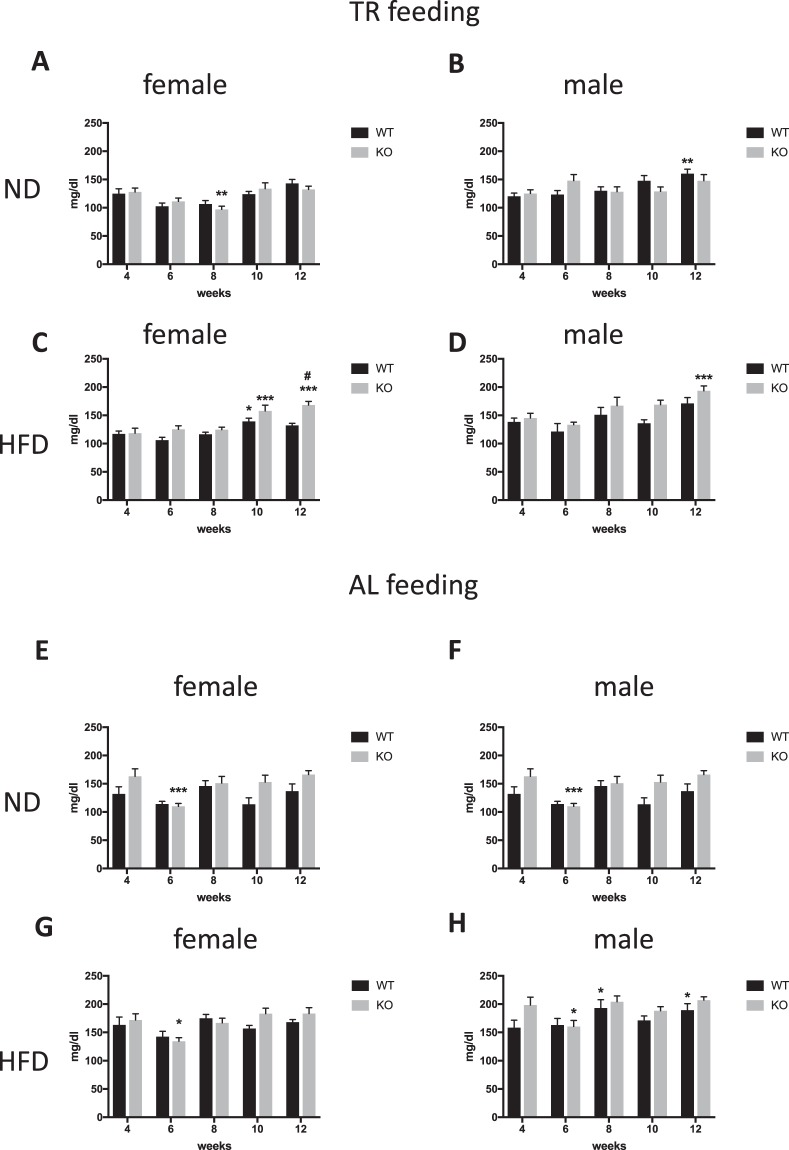
Blood glucose levels in mice kept on TR feeding protocol, receiving ND (females **A**, males **B**) or HFD (females **C**, males **D**) diet. Blood glucose levels of mice kept on AL feeding receiving ND is shown at panel **E** (females), **F** (males) or HFD at pane **G** (females) and **H** (males). *p < 0.05; **p < 0.01; ***p < 0.001 difference compared to week 3 from the same genotype according to Šidákś test. ^#^p < 0.05 difference between WT and KO according to Šidákś test. Bars represent mean values, whiskers SEM.

Analysis of blood glucose levels in female mice maintained on a TR ND showed a main effect of time (F_(4,52)_ = 6.518, p < 0.001), but no genotype effect and no interaction. However, post hoc analysis by Šidák’s multiple comparison test showed that only KO mice (week 8) had altered glucose levels at one time point (reduced compared to the first measurement at week 4) (Suppl. Table 4 and Fig. 3A). Similarly, in ND TR-fed group of male mice there was a significant time effect (F_(4,56)_ = 4.01, p = 0.006), but no genotype effect and no interaction were present. In males, the post hoc test showed a significant difference only in WT and again only at one time point (higher at week 12 compared to week 4) (Fig. 3B). KO females and males maintained on a ND had similar levels of blood glucose as their WT control groups in each time point.

Analysis of females maintained on a TR HFD showed a main genotype effect (F_(1,16)_ = 15.20, p = 0.001), time effect (F_(4,64)_ = 17.46, p < 0.0001), and an interaction (F_(4,64)_ = 2.525, p = 0.049). Post hoc analysis showed that KO females had significantly higher blood glucose levels than WT controls at week 12 (Fig. 3C). Blood glucose values were significantly increased at week 10 in WT and at week 10 and 12 in the KO compared to the baseline at week 4 (Fig. 3C). In male mice, there was a significant main time effect (F_(4,52)_ = 10.72, p < 0.0001) but no genotype effect (F_(1,13)_ = 4.438, p = 0.055) and no interaction (F_(4,52)_ = 0.6526, p = 0.6276). Post hoc analysis of animals maintained on a TR HFD showed a significant difference in the KO group at week 12 (Fig. 3D).

#### Blood glucose: AL feeding schedule

Female mice maintained on an AL ND showed a main genotype (F_(1,15)_ = 9.088 p = 0.008) and time effect (F_(4,60)_ = 4.595, p = 0.002). Post hoc analysis showed significantly higher blood glucose levels in KO female mice compared to WT controls at week 4 (Suppl. Table 4 and Fig. 3E). In males, a main time effect (F_(4,56)_ = 9.963, p < 0.0001) and interaction (F_(4,56)_ = 3.223, p = 0.0189), but no genotype effect (F_(1,14)_ = 2.859, p = 0.113) was observed. Post hoc analysis showed a significant time effect in KO males at week 5 (Fig. 3F).

Females maintained on an AL HFD showed a main time effect (F_(4,56)_ = 6.026, p = 0.0004), but no genotype effect (F_(1,14)_ = 1.051, p = 0.322) and no interaction (F_(4,56)_ = 1.535, p = 0.204) (Fig. 3G). Similarly, in males a main time effect was observed (F_(4,52)_ = 6.518, p = 0.0002), but no genotype effect (F_(1,13)_ = 2.108, p = 0.17) and no interaction (F_(4,52)_ = 1.606, p = 0.186) (Fig. 3H). Post hoc multiple comparison analysis in AL HFD female KO mice showed a significant reduction in glucose values at week 6 (Fig. 3G). In AL HFD KO group, males like females had reduced blood glucose levels at week 6, whereas WT males showed elevated levels at week 8 and 12 (Fig. 3H).

### Immunohistochemical analysis of hypothalamic peptides

#### Neuropeptide-Y

**Figure 4:**
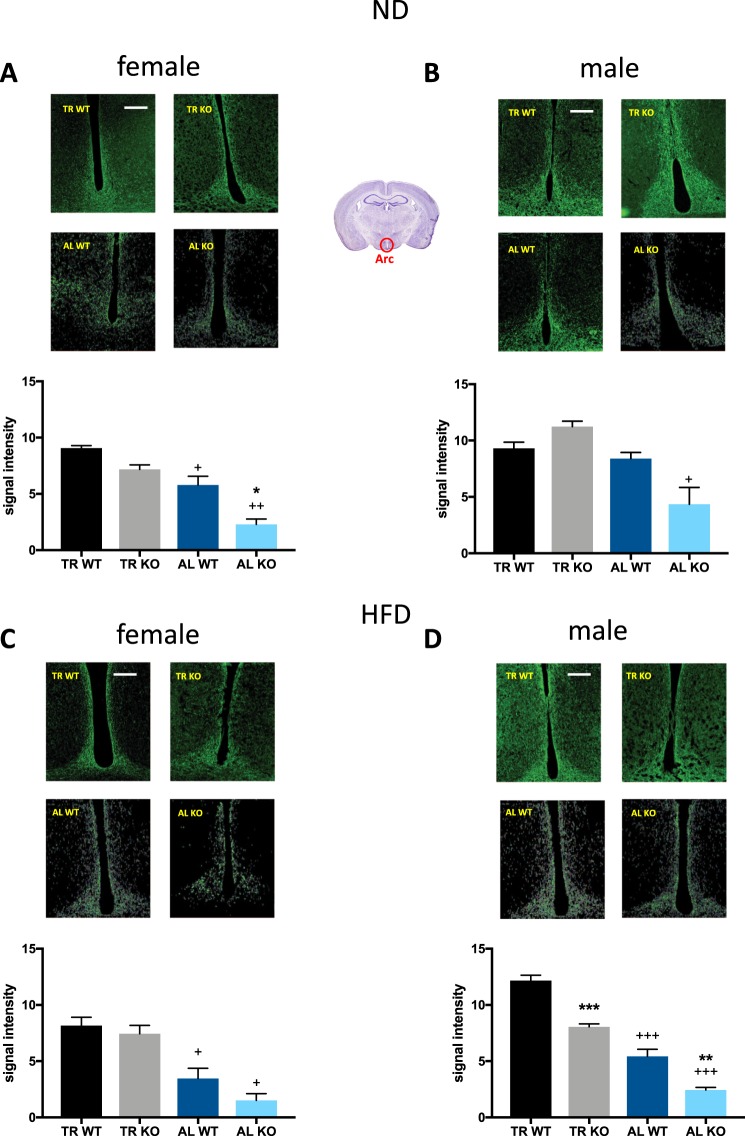
Immunohistochemical analysis of hypothalamic peptide NPY expression in the ARC. Region framed in red on the brain picture indicates the site of histological recordings. The mean signal intensity was calculated from sections from 3–4 mice per group. Female animals on a normal diet (ND) are shown in (**A**), males in (**B**). Female animals on a high fat diet (HFD) are shown in (**C**), males in (**D)**. The scale bar represents 250 µm. *p < 0.05, **p < 0.01, ***p < 0.001 difference between the genotypes having the same feeding schedule; ^+^p < 0.05, ^++^p < 0.01, ^+++^p < 0.0001 difference between the feeding schedule within the same genotypes, each using Šidákś test.

Analysis of neuropeptide-Y (NPY) levels in animals maintained on a ND revealed main genotype (F_(1,8)_ = 26.20, p = 0.0009) and feeding schedule effects (F_(1,8)_ = 11.54, p = 0.0094) in females, but no interaction (Fig. 4A). In males, a main effect was only observed for feeding schedule (F_(1,8)_ = 6.359, p = 0.0357), however not for genotype and no interaction was found (Fig. 4B). WT and KO animals on HFD showed a main feeding schedule effect (F_(1,7)_ = 19.16, p = 0.0032) in females, whereas in males, significant genotype (F_(1,8)_ = 54.34, p < 0.0001) and feeding schedule effects (F_(1,8)_ = 183 p < 0.0001) were seen (Fig. 4C,D). No significant interaction effect was observed. Post hoc analysis showed that female KO animals maintained on ND generally had lower NPY expression levels in AL feeding compared to their wild-type controls (Fig. 4A). In female ND group mice kept in AL had reduced NPY expression compared to mice in the TR group in both genotypes (Fig. 4A). The difference between TR and AL groups reached the level of significance in males only in KO mice (Fig. 4B). Females on HFD showed lower NPY expression levels in AL feeding as compared to TR feeding schedule in both genotypes (Fig. 4C). This effect was also observed in HFD-fed males (Fig. 4D). Additionally, KO males showed a lower NPY signal intensity as compared to WT males, in TR feeding as well as in AL feeding (Fig. 4D).

#### Orexin-A

**Figure 5:**
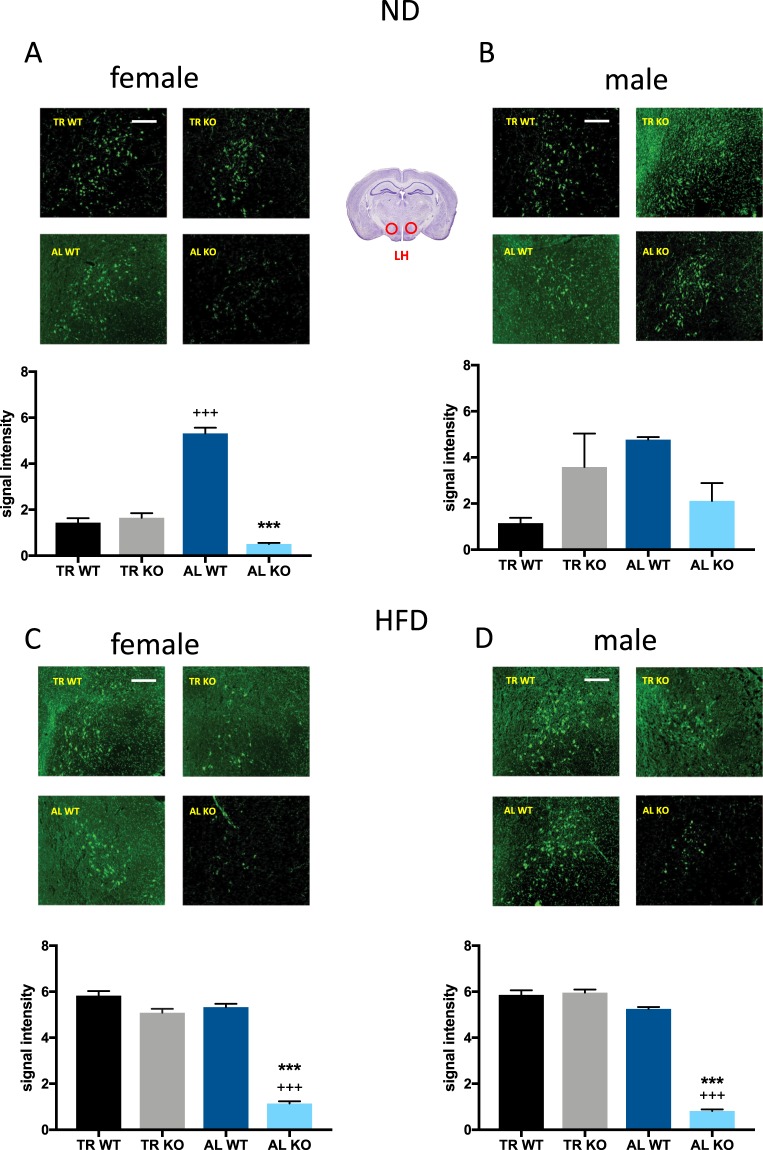
Immunohistochemical analysis of orexin-A expression in the lateral hypothalamus. Region framed in red on the brain picture indicates the site of histological recordings. The mean integrated signal density was calculated from sections from 3–4 mice per group. Female animals on normal diet (ND) are shown in (**A**), males in (**B**). Female animals on high fat diet (HFD) are shown in (**C**), males in (**D**). The scale bar represents 250 µm. ***p < 0.001 difference between the genotypes having the same feeding schedule ^+++^p < 0.001 difference between the feeding schedule within the same genotypes, each using Šidákś test.

Analysis of the hypothalamic peptide orexin-A in the lateral hypothalamic region of female mice maintained on a ND showed a main effect of genotype (F_(1,6)_ = 38.21, p = 0.0008), feeding schedule (F_(1,6)_ = 13.89, p = 0.0098) and interaction (F_(1,6)_ = 45.37, p = 0.0005) (Fig. 5A). In males, only an interaction effect (F_(1,7)_ = 7.032, p = 0.0329), but no genotype or feeding schedule effects were observed (Fig. 5B). In WT and KO animals maintained on HFD, a significant effect of feeding schedule (F_(1,8)_ = 135.4, p < 0.0001 in females and F_(1,8)_ = 257.6, p < 0.0001 in males), genotype effect (F_(1,8)_ = 166.1, p < 0.0001 in females and F_(1,8)_ = 149.1, p < 0.0001 in males) and interaction (F_(1,8)_ = 81.58, p < 0.0001 in females and (F_(1,8)_ = 169.1, p < 0.0001 in males) was found (Fig. 5C,D). Post hoc test of animals maintained on ND revealed that KO females had generally lower orexin-A expression levels in AL feeding group as compared to WT females (Fig. 5A). Additionally, WT females on AL feeding had higher signal density levels as compared to females on TR feeding schedule. KO animals on HFD on AL feeding schedule generally showed lower orexin-A expression compared to WT. This effect was seen in both sexes (Fig. 5C,D). Furthermore, KO males on AL feeding showed lower signal density levels of orexin peptide as compared to TR feeding group (Fig. 5D).

#### Relation between the level of orexigenic peptides NPY and orexin A, body weight gain and blood glucose

Lastly, we asked how NPY and orexin A influences body weight gain and blood glucose levels. As shown in Table 1 the level of NPY in the arcuate nucleus and orexin A in the lateral hypothalamus showed a strong, significant correlation (p = 0.005). Interestingly, NPY but not orexin A negatively correlated with body weight gain (p = 0.022) and blood glucose values (p = 0.045) meaning that animals with high NPY staining intensity had lower body weight and lower blood glucose at the end of the experiment.

**Table 1:** Correlation between NPY, orexin A, body weight and blood sugar at week 12.

	Orexin	NPY	Body weight change	Blood sugar
Orexin		**0**.**418**	0.115	−0.217
NPY	**0**.**418**		**−0**.**357**	**−0**.**315**
Body weight change	0.115	−0.357		−0.095
Blood sugar	−0.217	**−0**.**315**	−0.095	

Pearson r values are shown, significant values are highlighted as bold.

#### Immunohistochemical analysis of the effect of HFD on the expression of KOR and Pdyn

**Figure 6:**
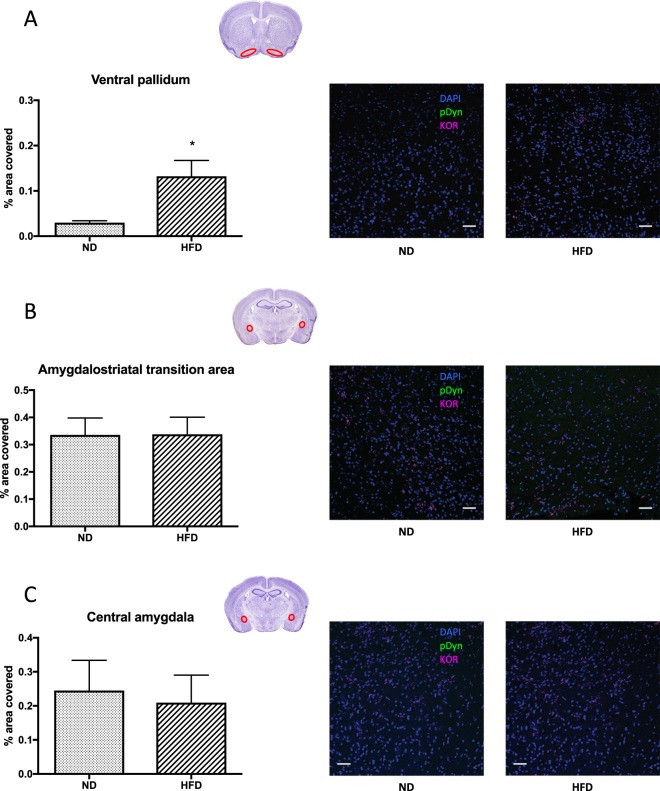
Immunohistochemical analysis of KOR and Pdyn expression. Region framed in red on the brain image indicates the site of histological analysis. The mean integrated signal density shown at the left panels was calculated from 3–4 male wild-type mice per group (average of 4–6 sections per animal), right panels show representative images. The scale bar represents 50 µm. *p < 0.05 difference between the ND and HFD groups using Studentś t-test.

Lastly, we analysed how HFD influences dynorphin signaling, because the KO mice had a much stronger phenotype in the HFD than in the ND groups. For this experiment, we used only male mice, because the phenotype difference was more pronounced in this sex. Generally, a Pdyn signal was only marginally detectable in the insular cortex and practically undetectable in 24 other brain areas related to feeding behaviour (Table 2). KOR was present in three areas, in the central amygdala, in the amygdalostriatal transition area and in the ventral pallidum (Table 2). HFD significantly increased the expression of KOR in the ventral pallidum (t_6_ = 3.007; p = 0.024), but not in the central amygdala (t_8_ = 0.297; p = 0.774) or in the amygdalostriatal transition area (t_7_ = 0.025; p = 0.981) (Fig. 6).

**Table 2:** Effect of HFD on KOR and Pdyn expression in the brain. Regions indicated with bold show expression above the detection level. - indicates no expression, + marginal expression and +++ significant expression.

Brain region	Position (Bregma, mm)	KOR	Pdyn
PreL, MO, VO, LO, Pir	2.68–1.98	−	−
**AID**, **AIV**, PreL, Cg	2.34–1.10	−	**+**
AcbSh, AcbC, PreL, IL, Cg, Pir	1.94–1.10	−	−
**VP**, **Tu**	**1**.**94–0**.**38**	**+++**	−
BLA, **CeA**, **Ast**, PVN	**−0**.**94–1**.**82**	**+++**	−
DG, CA1, LHA, Arc	−1.22–1.82	−	−

PreL- prelimbic cortex; MO- medial orbitofrontal cortex; VO- ventral orbitofrontal cortex; LO- lateral orbitofrontal cortex; Pir- piriform cortex; AID- agranular insular cortex, dorsal; AIV- agranular insular cortex, ventral; PreL- prelimbic cortex; Cg- cingulate cortex; AcbSh- accumbens nucleus, shell; AcbC- accumbens nucleus, core; IL- infralimbic cortex; VP- ventral pallidum; Tu- olfactory tubercle; BLA- basolateral amygdaloid nucleus, lateral part; CeA- central amygdaloid nucleus; Ast- amygdalostriatal transition area; PVN- paraventricular hypothalamic nucleus; DG- dentate gyrus; CA1, CA3 –field CA1, CA3 of the hippocampus; LHA- lateral hypothalamic area; Arc- arcuate hypothalamic nucleus.

## Discussion

In the current study, we exposed Dyn KO mice to two different diets (ND, HFD) under two different feeding regimens (AL, TR). An overview of the results is shown in Table 3. The major finding of this study was that 1; Dyn KO mice showed a significantly enhanced weight gain during the experiments especially in the HFD groups, suggesting a specific role of dynorphin regulation of weight gain. 2; This increased weight gain was not explained by hyperphagia or altered glucose metabolism, because Dyn KO mice did not eat more and their blood sugar level was increased only during one time point in ND AL and HFD TR females. 3; The hypothalamic levels of the orexigenic peptides NPY and orexin in overweight male Dyn KO mice kept on HFD were decreased as expected. Importantly, reduced food intake did not lead to lower body weight gain suggesting that Dyn signaling is necessary for coupling changes in energy intake to the body weight.

**Table 3:** Overview of the differences between WT and KO.

Diet	Sex	Regime	Weight gain	Food intake	Blood glucose	NPY	Orexin
ND	female	AL	normal	**decreased**	**increased**	**decreased**	**decreased**
		TR	normal	normal	normal	normal	normal
	male	AL	normal	normal	normal	normal	normal
		TR	**increased**	normal	normal	normal	normal
HFD	female	AL	normal	normal	normal	normal	**decreased**
		TR	**increased**	normal	**increased**	normal	normal
	male	AL	**increased**	normal	normal	**decreased**	**decreased**
		TR	**increased**	normal	normal	**decreased**	normal

Normal: no statistical difference between WT and KO. Increased: significantly higher values in KO compared to WT. Decreased: significantly lower values in KO compared to WT.

AL feeding resulted in a more pronounced increase in body weight than TR feeding, although animals on AL consumed similar amounts of food. The difference in body weight was particularly prominent in animals maintained on HFD. Dyn KO animals, especially male mice, were significantly heavier than their WT controls. The resulting weight pattern was in the following order ranging from low to high: WT TR < KO TR < WT AL < KO AL. This pattern was inversely correlated to the level of hypothalamic NPY expression. Our study suggests that Dyn modulates metabolic changes associated with diet composition and feeding regimen, which is more pronounced in the male animals.

The body weight data revealed that constitutive deletion of Dyn increased the body weight gain more when the animals were maintained on a HFD than on ND. Among KO mice kept on ND, only males on TR feeding regimen had higher weight gain than WT controls. The weight gain of KO in the HFD group significantly exceeded that of WT controls in females on TR as well as males both on AL or TR. Our result now suggests that Dyn limits weight gain, and this effect is especially important in case of exposure to highly caloric palatable foods. Although this result is in good agreement with the known role of Dyn signaling in terminating hedonic behaviour^27^, it contradicts the findings of a previous study, in which Dyn deletion did not affect body weight in neither ND nor HFD conditions^28^. The discrepancies may be due to the differences in HFD composition (46% fat, 4.72 kcal/g versus 59% fat, 5.49 kcal/g used in our study) and the age of animals at the beginning of the feeding regimen (7 versus 12 weeks old). The body weight gain of our animals is in good agreement with other studies where animals were exposed to a similar diets and feeding conditions^29^.

Other studies on WT mice maintained on a HFD using a TR feeding regimen have shown that the animals consumed the same amount of calories as the AL feeding group, protecting them from weight gain and obesity. In addition, the frequency of feeding episodes, rather than the meal size or total energy intake, contribute to weight gain and development of obesity^29–31^. It is important to note, that the different consistency and therefore different spillage between the softer HFD and harder ND pellets may have influenced the calculated amount of food. However, the contribution of spillage to the total amount of food is probably less than 5% (otherwise it could not remain unnoticed) therefore this potential bias has only a minor effect on our calculations.

Our results show that animals maintained on a TR feeding regimen had lower body weights compared to the AL feeding group, which supports the previous findings^29–31^. This effect was observed in all groups of animals, particularly in those which were maintained on a HFD. Interestingly, a human study that assessed the effect of the TR food intake in overweight individuals showed results similar to ours. The participants were restricted to eating only within a self-selected 10–11-hour period every other day^32^. On the alternating days, they were permitted to consume food AL. The outcome showed that the participants lost 4% of their body weight in 16 weeks, and retained this weight loss for up to 1 year. Alternatively, it was shown that intermittent feeding in humans affects a wide range of metabolic markers, risk factors and diseases, including body fat and blood pressure after 2–3 weeks of alternate day fasting^33–35^. Furthermore, the time of food access has a direct effect on body weight regulation, and as in our results, this effect was seen particularly in the HFD group.

In general, we observed significantly higher body weights in Dyn KO male mice compared to female mice in the HFD feeding regimen. Furthermore, studies on sex-specific effects in body weight regulation have reported that HFD-fed males were more susceptible to weight gain compared to females with similar energy intake. These alterations in body weight gain could be caused either by reduced metabolic rate or the anti-obesity effects of estrogen in females through estrogen receptor α, as reported earlier^35–37^.

It is of note that Dyn is generated by proteolytic cleavage from the precursor protein prodynorphin (Pdyn) together with other biologically active peptides like neo-endorphins and leumorphine^38^. Thus, in our KO animals not only Dyn but also other Pdyn-derived peptides and their metabolites are depleted. Although Pdyn-derived peptides generally bind and activate kappa opioid receptors, leumorphin has other binding site(s) in addition to that for the kappa opioid receptor^39^. Thus, using Dyn KO animals we cannot exclude that some of the observed effects are only partially mediated by the kappa opioid receptor.

Our study suggests that Dyn influences feeding behavior and metabolism, but it is not fully known how diet influences Dyn signalling. Food deprivation, which the animals experienced in the TR group in the first week(s) until adapting to the new feeding regime, increased Dyn and kappa opioid receptor expression in the spinal cord^40^ but not in the brain^41^. Our study now suggests that HFD increases Dyn signaling by increasing kappa opioid receptor levels in one of the most important hedonic hotspots responding to mu opiod stimulation, in the ventral pallidum, an area that is also interconnected with hypothalamic nuclei that regulate feeding^42^. The endogenous opioid system is involved in the homeostatic control of feeding by regulating the release of orexigenic hypothalamic peptides such as NPY and orexin-A. The activities of these hypothalamic peptides are dependent on opioid signalling^43^. It is known that Dyn and NPY are important players in modulation of energy homeostasis and neuroendocrine regulation^18^. The hypothalamic ARC is the major site of expression for NPY within neurons that project to the paraventricular nucleus, dorsomedial and lateral hypothalamus (LH) and other hypothalamic sites. NPY can exert diverse effects on behavior and other functions; the most noticeable of them is the stimulation of feeding after central administration^44^. In Dyn KO animals, irrespective of diet type, the NPY expression in the ARC was prominently lower if they were maintained on AL food compared to TR feeding. This implies that feeding regimen, especially the duration of food consumption, plays a crucial role in the regulation of NPY. Indeed, our results showed that high NPY levels were associated with low body weight gain and reduced blood glucose levels. Although NPY and orexin A levels were closely correlated, we could not detect statistically significant correlation between the intensity of orexin A immunoreactivity and body weight gain or blood glucose level.

Orexins, in general, are appetite-stimulating neuropeptides that regulate body weight homeostasis^45^. Neurons expressing the orexin-A peptide are well known to populate the lateral aspects of the hypothalamus^19,46^. Orexigenic neurons in the LH are also known to play a role in arousal, reward seeking and feeding behavior^47^. Our results from orexin-A peptide immunostaining show that the peptide levels in Dyn KO mice during AL feeding regimen were significantly lower compared to the TR feeding group. These results correspond well with our NPY results, indicating that both of the orexigenic peptides are upregulated in TR fed mice. Importantly, the reduced levels of orexigenic peptides in KO kept on HFD did not reduce their food intake and body weight, which suggests an important role of Dyn in body weight control, especially when eating highly palatable and calorie-dense food.

We conclude that lack of Dyn causes uncoupling of body weight gain from food intake in animals when maintained on a HFD. Additionally, there is a reduction in levels of hypothalamic peptides NPY and orexin-A in Dyn KO. Our study suggests that Dyn modulates metabolic changes associated with HFD consumption and a TR feeding regimen. We also demonstrate that HFD feeding increases KOR levels specifically in ventral pallidum. Further studies with kappa opioid receptor agonists and antagonists should clarify whether disproportionate gain of body weight on highly caloric foods could be influenced by pharmacological manipulation of kappa opioid receptors and answer whether agonists could be used as a potential treatment strategy against obesity in overweight patients.

## Supplementary information

Supplementary dataset 1.

## References

[1] Wang Chao-Yung, Liao James K. (2011). A Mouse Model of Diet-Induced Obesity and Insulin Resistance. Methods in Molecular Biology.

[2] Spanagel, R. & Weiss, F. The dopamine hypothesis of reward: past and current status. *Trends Neurosci.* **22**, 521–527 (1999). 10.1016/s0166-2236(99)01447-2 10529820

[3] South T, Huang X-F (2008). High-Fat Diet Exposure Increases Dopamine D2 Receptor and Decreases Dopamine Transporter Receptor Binding Density in the Nucleus Accumbens and Caudate Putamen of Mice. Neurochem. Res..

[4] Hayward M, Schaichborg A, Pintar J, Low M (2006). Differential involvement of endogenous opioids in sucrose consumption and food reinforcement. Pharmacol. Biochem. Behav..

[5] Arjune D, Bodnar RJ (1990). Suppression of nocturnal, palatable and glucoprivic intake in rats by the κ opioid antagonist, nor-binaltorphamine. Brain Res..

[6] Drewnowski A, Krahn D, Demitrack M, Nairn K, Gosnell B (1992). Taste responses and preferences for sweet high-fat foods: Evidence for opioid involvement☆. Physiol. Behav..

[7] Pecina S (2005). Hedonic Hot Spot in Nucleus Accumbens Shell: Where Do -Opioids Cause Increased Hedonic Impact of Sweetness?. J. Neurosci..

[8] Schwarzer C (2009). 30 years of dynorphins — New insights on their functions in neuropsychiatric diseases. Pharmacol. Ther..

[9] Steiner H, Gerfen CR (1998). Role of dynorphin and enkephalin in the regulation of striatal output pathways and behavior. Exp. Brain Res..

[10] Carlezon WA (1998). Regulation of Cocaine Reward by CREB. Science (80-.)..

[11] Williams G, Harrold JA, Cutler DJ (2000). The hypothalamus and the regulation of energy homeostasis: lifting the lid on a black box. Proc. Nutr. Soc..

[12] Yu JH, Kim M-S (2012). Molecular Mechanisms of Appetite Regulation. Diabetes Metab. J..

[13] Clark JT, Kalra PS, Crowley WR, Kalra SP (1984). Neuropeptide Y and human pancreatic polypeptide stimulate feeding behavior in rats. Endocrinology.

[14] Sakurai T (1998). Orexins and Orexin Receptors: A Family of Hypothalamic Neuropeptides and G Protein-Coupled Receptors that Regulate Feeding Behavior. Cell.

[15] Kokot F, Ficek R (1999). Effects of Neuropeptide Y on Appetite. Miner. Electrolyte Metab..

[16] Bi S, Robinson BM, Moran TH (2003). Acute food deprivation and chronic food restriction differentially affect hypothalamic NPY mRNA expression. Am. J. Physiol. - Regul. Integr. Comp. Physiol..

[17] Przewłocki R, Gramsch C, Pasi A, Herz A (1983). Characterization and localization of immunoreactive dynorphin, α-neo-endorphin, met-enkephalin and substance P in human spinal cord. Brain Res..

[18] Lin S (2006). Distribution of prodynorphin mRNA and its interaction with the NPY system in the mouse brain. Neuropeptides.

[19] Date Y (1999). Orexins, orexigenic hypothalamic peptides, interact with autonomic, neuroendocrine and neuroregulatory systems. Proc. Natl. Acad. Sci..

[20] Horvath TL, Diano S, Van Den Pol AN (1999). Synaptic interaction between hypocretin (Orexin) and neuropeptide Y cells in the rodent and primate hypothalamus. J. Neurosci..

[21] Edwards CMB (1999). The effect of the orexins on food intake: Comparison with neuropeptide Y, melanin-concentrating hormone and galanin. J. Endocrinol..

[22] Haynes AC (2000). A selective orexin-1 receptor antagonist reduces food consumption in male and female rats. Regul. Pept..

[23] Hara J (2001). Genetic Ablation of Orexin Neurons in Mice Results in Narcolepsy, Hypophagia, and Obesity. Neuron.

[24] Valdivia S, Patrone A, Reynaldo M, Perello M (2014). Acute High Fat Diet Consumption Activates the Mesolimbic Circuit and Requires Orexin Signaling in a Mouse Model. PLoS One.

[25] Muschamp JW (2014). Hypocretin (orexin) facilitates reward by attenuating the antireward effects of its cotransmitter dynorphin in ventral tegmental area. Proc. Natl. Acad. Sci..

[26] Zimmer A (2001). Absence of Δ-9-tetrahydrocannabinol dysphoric effects in dynorphin-deficient mice. J. Neurosci..

[27] Le Merrer J, Becker JAJ, Befort K, Kieffer BL (2009). Reward Processing by the Opioid System in the Brain. Physiol. Rev..

[28] Sainsbury A (2007). Dynorphin Knockout Reduces Fat Mass and Increases Weight Loss during Fasting in Mice. Mol. Endocrinol..

[29] Hatori M (2012). Time-Restricted Feeding without Reducing Caloric Intake Prevents Metabolic Diseases in Mice Fed a High-Fat Diet. Cell Metab..

[30] Chaix A, Zarrinpar A, Miu P, Panda S (2014). Time-Restricted Feeding Is a Preventative and Therapeutic Intervention against Diverse Nutritional Challenges. Cell Metab..

[31] Murphy M, Mercer J (2014). Intermittent Feeding Schedules—Behavioural Consequences and Potential Clinical Significance. Nutrients.

[32] Gill S, Panda S (2015). A Smartphone App Reveals Erratic Diurnal Eating Patterns in Humans that Can Be Modulated for Health Benefits. Cell Metab..

[33] Halberg N (2005). Effect of intermittent fasting and refeeding on insulin action in healthy men. J. Appl. Physiol..

[34] Heilbronn LK, Smith SR, Martin CK, Anton SD, Ravussin E (2005). Alternate-day fasting in nonobese subjects: Effects on body weight, body composition, and energy metabolism. Am. J. Clin. Nutr..

[35] Gao Q (2007). Anorectic estrogen mimics leptin’s effect on the rewiring of melanocortin cells and Stat3 signaling in obese animals. Nat. Med..

[36] Hwang L-L (2010). Sex Differences in High-fat Diet-induced Obesity, Metabolic Alterations and Learning, and Synaptic Plasticity Deficits in Mice. Obesity.

[37] Winzell MS, Ahren B (2004). The High-Fat Diet-Fed Mouse: A Model for Studying Mechanisms and Treatment of Impaired Glucose Tolerance and Type 2 Diabetes. Diabetes.

[38] Suda M (1983). A novel opioid peptide, leumorphin, acts as an agonist at the κ opiate receptor. Life Sci..

[39] Lee BD (2005). Leumorphin has an anti-apoptotic effect by activating epidermal growth factor receptor kinase in rat pheochromocytoma PC12 cells. J. Neurochem..

[40] De Los Santos-Arteaga M, Sierra-Domínguez SA, Fontanella GH, Delgado-García JM, Carrión ÁM (2003). Analgesia Induced by Dietary Restriction Is Mediated by the Kappa-Opioid System. J. Neurosci..

[41] Barnes MJ, Primeaux SD, Bray GA (2008). Food deprivation increases the mRNA expression of μ-opioid receptors in the ventral medial hypothalamus and arcuate nucleus. Am. J. Physiol. Integr. Comp. Physiol..

[42] Castro, D. C., Cole, S. L. & Berridge, K. C. Lateral hypothalamus, nucleus accumbens, and ventral pallidum roles in eating and hunger: interactions between homeostatic and reward circuitry. *Front. Syst. Neurosci*. **9** (2015). 10.3389/fnsys.2015.00090 PMC4466441 26124708

[43] Sweet DC, Levine AS, Kotz CM (2004). Functional opioid pathways are necessary for hypocretin-1 (orexin-A)-induced feeding. Peptides.

[44] Wisialowski T (2000). Adrenalectomy reduces neuropeptide Y–induced insulin release and NPY receptor expression in the rat ventromedial hypothalamus. J. Clin. Invest..

[45] Sakurai T (2014). The role of orexin in motivated behaviours. Nat. Rev. Neurosci..

[46] Yamamoto Y (1999). Down regulation of the prepro-orexin gene expression in genetically obese mice. Mol. Brain Res..

[47] Harris GC, Wimmer M, Aston-Jones G (2005). A role for lateral hypothalamic orexin neurons in reward seeking. Nature.

