# Supplementary material for: Modulation of feeding behavior and metabolism by dynorphin
Source: Sci Rep. 2020 Mar 2;10:3821. doi: 10.1038/s41598-020-60518-0 (PMC7052232; doi:10.1038/s41598-020-60518-0)

Modulation of feeding behavior and metabolism by dynorphin

Aishwarya Ghule[1], Ildiko Rácz[1,2], Andras Bilkei-Gorzo[1], Este Leidmaa[1], Meike Sieburg[3] and Andreas Zimmer[1]

[1] Institute of Molecular Psychiatry, University of Bonn, Medical Faculty, Venusberg Campus 1, 53127 Bonn, Germany

[2] Present address: Department of Neurodegenerative Diseases & Geriatric Psychiatry University of Bonn, Medical Faculty, Venusberg-Campus 1, 53127 Bonn, Germany

[3] Present address: Aarhus University, Department of Biomedicine/DANDRITE Capogna group, Ole Worms Alé 6, 8000, Aarhus C, Denmark

Correspondence should be addressed to ABG (Email address: abilkei@uni-bonn.de)

Supplementary Table 1 Regression analysis of body weight changes

Female ND

	WT AL	WT TR	KO AL	KO TR
Slope	0.2495 ± 0.01723	0.3207 ± 0.03104	0.2691 ± 0.01376	0.3212 ± 0.02239
Goodness of Fit (R square)	0.9633	0.9302	0.9795	0.9626
Is slope different from zero?	F 1,8 = 209.8	F 1,8 = 106.7	F 1,8 = 382.4	F 1,8 = 205.7
P value	<0.0001	<0.0001	<0.0001	<0.0001
Are slopes equal?	F 3,32 =2.725; p = 0.0604			

Male ND

	WT AL	WT TR	KO AL	KO TR
Slope	0.2409 to 0.4153	0.3123 to 0.3752	0.21 to 0.3849	0.5211 to 0.6034
Goodness of Fit (R square)	0.904	0.9876	0.8849	0.992
Is slope different from zero?	F 1,8 = 75.29	F 1,8 = 636.0	F 1,8 = 61.48	F 1,8 = 992.1
P value	<0.0001	<0.0001	<0.0001	<0.0001
Are slopes equal?	F 3,32 =17.40; p < 0.001			

Female HFD

	WT AL	WT TR	KO AL	KO TR
Slope	1.703 ± 0.03322	0.4158 ± 0.02269	1.975 ± 0.05399	1.065 ± 0.07628
Goodness of Fit (R square)	0.997	0.9767	0.9941	0.9606
Is slope different from zero?	F 1,8 = 2629	F 1,8 = 335.8	F 1,8 = 1338	F 1,8 = 195.1
P value	<0.0001	<0.0001	<0.0001	<0.0001
Are slopes equal?	F 3,32 =187.4; p < 0.001			

Male HFD

	WT AL	WT TR	KO AL	KO TR
Slope	1.07 to 1.182	0.6355 to 0.7805	1.991 to 2.271	1.052 to 1.235
Goodness of Fit (R square)	0.9963	0.9845	0.9935	0.9905
Is slope different from zero?	F 1,8 = 2132	F 1,8 = 507.2	F 1,8 = 1232	F 1,8 = 992.1
P value	<0.0001	<0.0001	<0.0001	<0.0001
Are slopes equal?	F 3,32 =213.4; p < 0.001			

Supplementary Table 2 Three-way ANOVA analysis of body weight values

Female ND

**Source of variation**	**Df**	**Sum of squares**	**Mean square**	**F**	**P value**
Genotype	1	3.7135	3.7135	0.16969	0.683313
Administration	1	154.3538	154.3538	7.05334	0.017757
Genotype x administration	1	28.1493	28.1493	1.28631	0.265711
Error	30	656.5137	21.8838		
Time	10	312.0365	49.2937	72.49239	0.000000
Genotype x time	10	2.0577	0.2058	0.47804	0.903777
Administration x time	10	7.3549	0.7355	1.70868	0.078032
Genotype x administration x time	10	2.6234	0.2623	0.60948	0.805616
Error	300	129.1321	0.4304		

Male ND

**Source of variation**	**Df**	**Sum of squares**	**Mean square**	**F**	**P value**
Genotype	1	33.6678	33.6678	1.91350	0.177506
Administration	1	120.1119	120.1119	6.82653	0.014283
Genotype x administration	1	111.6486	111.6486	6.34552	0.017757
Error	28	492.6566	17.5949		
Time	10	492.9366	49.2937	46.78112	0.000000
Genotype x time	10	12.4798	1.2480	1.18437	0.301116
Administration x time	10	19.8644	1.9864	1.88519	0.047054
Genotype x administration x time	10	23.5026	2.3503	2.23046	0.016351
Error	280	295.0384	1.0537		

Female HFD

**Source of variation**	**Df**	**Sum of squares**	**Mean square**	**F**	**P value**
Genotype	1	177.78	177.78	3.7778	0.061370
Administration	1	6387.30	6387.30	135.7250	0.000000
Genotype x administration	1	13.47	13.47	0.2862	0.596585
Error	30	1411.82	47.06		
Time	10	5778.65	577.86	343.3111	0.000000
Genotype x time	10	191.79	19.18	11.3944	0.000000
Administration x time	10	1177.49	117.75	69.9550	0.000000
Genotype x administration x time	10	33.64	3.36	0.60948	0.033247
Error	300	504.96	1.68		

Male HFD

**Source of variation**	**Df**	**Sum of squares**	**Mean square**	**F**	**P value**
Genotype	1	1200.69	1200.69	14.1087	0.000880
Administration	1	2956.28	2956.28	34.7378	0.000003
Genotype x administration	1	346.70	346.70	4.0739	0.053978
Error	26	2212.67	85.10		
Time	10	5241.09	524.11	297.5148	0.000000
Genotype x time	10	413.51	41.35	23.4730	0.000000
Administration x time	10	474.10	47.41	26.9125	0.000000
Genotype x administration x time	10	79.81	7.98	4.5302	0.000006
Error	260	458.02	1.76		

Supplementary Table 3 Three-way ANOVA analysis of food consumed

Female ND

**Source of variation**	**Df**	**Sum of squares**	**Mean square**	**F**	**P value**
Genotype	1	3459	3459	0.815	0.417791
Administration	1	809039	809039	190.5	0.000160
Genotype x administration	1	69839	69839	16.45	0.015403
Error	4	16983	4246		
Time	11	82149	7468	0.899	0.548835
Genotype x time	11	46215	4201	0.506	0.889109
Administration x time	11	52705	4791	0.577	0.837120
Genotype x administration x time	11	36113	3283	0.395	0.950740
Error	44	365556	8308		

Male ND

**Source of variation**	**Df**	**Sum of squares**	**Mean square**	**F**	**P value**
Genotype	1	94429	94429	1.213	0.332469
Administration	1	120785	120785	1.552	0.280800
Genotype x administration	1	77330	77330	0.994	0.375246
Error	4	311262	77816		
Time	11	133157	12105	3.785	0.000745
Genotype x time	11	13347	1213	0.379	0.957361
Administration x time	11	23333	2121	0.663	0.764431
Genotype x administration x time	11	42219	3838	1.200	0.315233
Error	44	140724	3198		

Female HFD

**Source of variation**	**Df**	**Sum of squares**	**Mean square**	**F**	**P value**
Genotype	1	1445	1445	0.154	0.714465
Administration	1	940220	940220	100.4	0.000557
Genotype x administration	1	9799	9799	1.047	0.364118
Error	4	37449	9362		
Time	11	396322	36029	10.443	0.000000
Genotype x time	11	15720	1429	0.414	0.941983
Administration x time	11	65787	5981	1.734	0.097214
Genotype x administration x time	11	32072	2916	0.845	0.597621
Error	44	151801	3450		

Male HFD

**Source of variation**	**Df**	**Sum of squares**	**Mean square**	**F**	**P value**
Genotype	1	4644	4644	0.591	0.484959
Administration	1	309886	309886	39.431	0.003284
Genotype x administration	1	588	588	0.075	0.798067
Error	4	31436	7859		
Time	11	210759	19160	6.721	0.000002
Genotype x time	11	14601	1327	0.466	0.914436
Administration x time	11	19003	1728	0.606	0.813508
Genotype x administration x time	11	6952	632	0.222	0.994947
Error	44	125440	2851		

Supplementary Table 4: Blood glucose levels in WT and KO animals

Male ND	TR WT<br>mean mg/dL	TR KO<br>mean mg/dL	AL WT<br>mean mg/dL	AL KO<br>mean mg/dL
week 3	120.37 ± 9.7	125.25 ± 9.79	132.11 ± 9.23	163.14 ± 10.46
week 5	123.5 ± 7.66	148 ± 7.67	114.33 ± 7.23	110.14 ± 8.2
week 7	130.12 ± 8.9	128.5 ± 8.9	146.11 ± 8.39	151.14 ± 9.51
week 9	147.75 ± 8.45	129 ± 8.46	113.66 ± 7.97	153 ± 9.04
week 11	160.37 ± 8.66	147.75 ± 8.66	137 ± 8.17	166.28 ± 9.26

Female ND	TR WT<br>mean mg/dL	TR KO<br>mean mg/dL	AL WT<br>mean mg/dL	AL KO<br>mean mg/dL
week 3	125.12 ± 9.79	128 ± 9.23	107 ± 9.23	136.87 ± 9.79 *
week 5	102.62 ± 7.67	111.44 ± 7.23	91.55 ± 7.23	111.87 ± 7.67
week 7	106.62 ± 8.9	97.22 ± 8.39	118.55 ± 8.39	133.75 ± 8.9
week 9	124 ± 8.46	133.77 ± 7.97	130.66 ± 7.97	124.12 ± 8.46
week 11	143.25 ± 8.66	132.55 ± 8.17	112.88 ± 8.17	139 ± 8.66

Male HFD	TR WT	TR KO	AL WT	AL KO
	mean mg/dL	mean mg/dL	mean mg/dL	mean mg/dL
week 3	138.42 ± 10.46	145.25 ± 9.79	158.5 ± 9.79	198.42 ± 10.46
week 5	121.42 ± 8.2	133.5 ± 7.67	163.12 ± 7.67	160.57 ± 8.2
week 7	150.85 ± 9.51	167.25 ± 8.9	193.12 ± 8.9	204.28 ± 9.51
week 9	135.85 ± 9.04	168.87 ± 8.46	171.37 ± 8.46	188.42 ± 9.04
week 11	171.14 ± 9.26	193.5 ± 8.66	189.37 ± 8.66	207.14 ± 9.26

Female HFD	TR WT	TR KO	AL WT	AL KO
	mean mg/dL	mean mg/dL	mean mg/dL	mean mg/dL
week 3	117.1 ± 8.76	118.37 ± 9.79	163.5 ± 9.79	171.87 ± 9.79
week 5	106.1 ± 6.86	125.25 ± 7.67	142.5 ± 7.67	134.5 ± 7.67
week 7	116.5 ± 7.96	124.62 ± 8.9	175.12 ± 8.9	166.87 ± 8.9
week 9	139.3 ± 7.56	157.75 ± 8.46	156.87 ± 8.46	183.25 ± 8.46
week 11	132.2 ± 7.75	168.12 ± 8.66 *	168.12 ± 8.66	183.37 ± 8.66

Supplementary Figure S1. Body weight values at the beginning of the experiments. *** p < 0.001 difference between males and females according to Šidák´s test. # p < 0.05 difference between WT and KO according to Šidák´s test. Bars represent mean values, whiskers SEM.

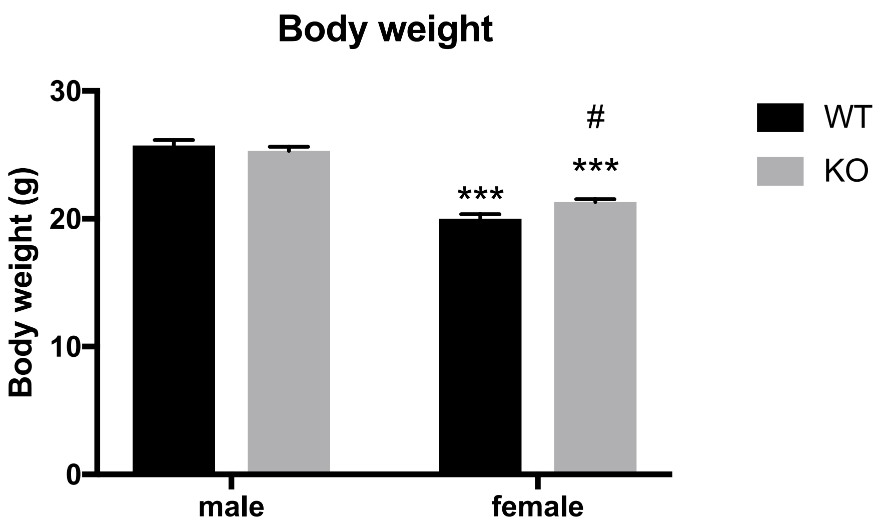

Supplementary Figure S2. High magnification images of immunohistochemical staining of NPY in the arcuate nucleus. Female animals on a normal diet are shown in (**A**), males in (**B**). Female animals on a high fat diet are shown in (**C**), males in (**D**). The scale bar represents 50 µm

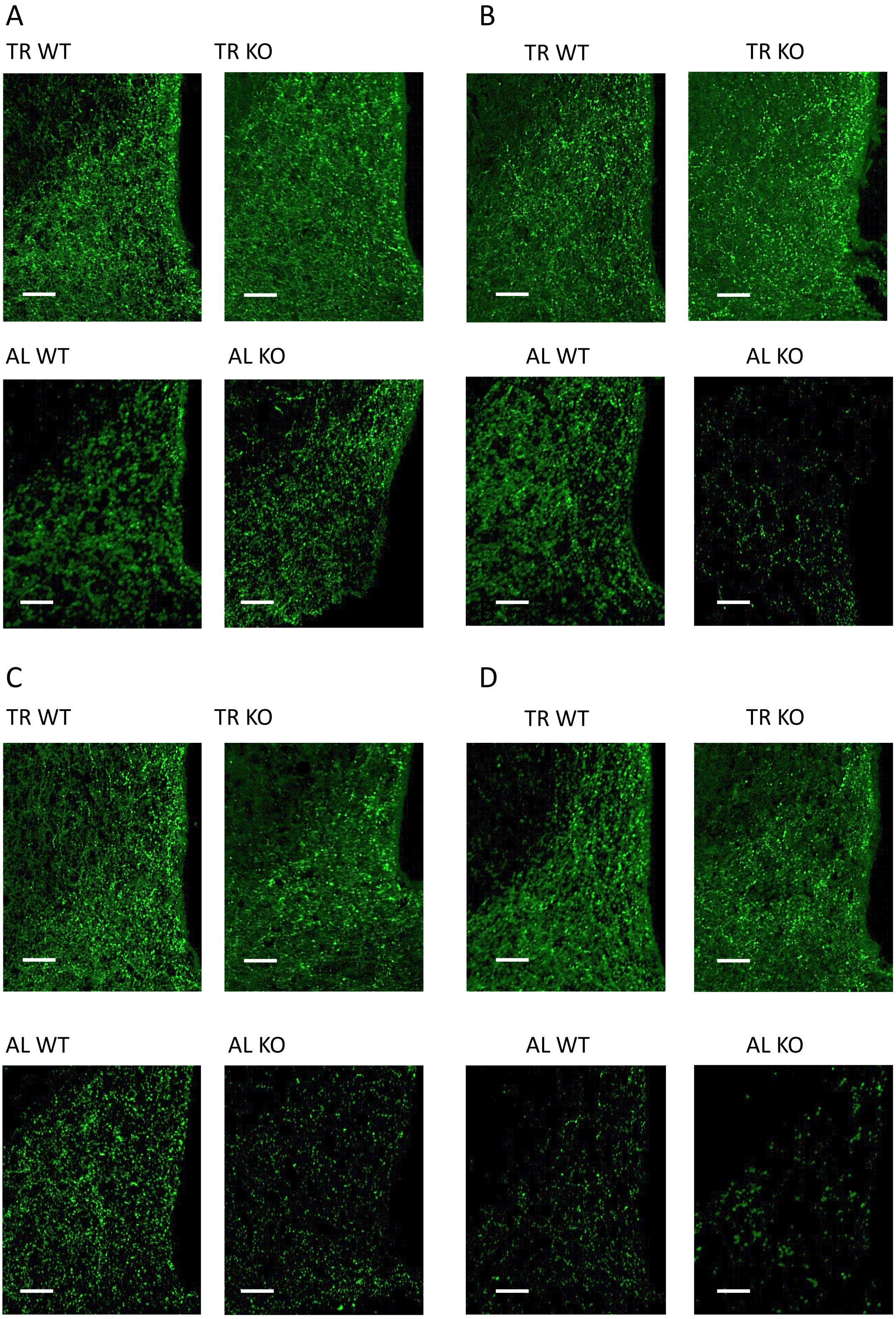

Supplementary Figure S3. High magnification images of immunohistochemical staining of orexin-A in the lateral hypothalamus. Female animals on a normal diet are shown in (**A**), males in (**B**). Female animals on a high fat diet are shown in (**C**), males in (**D)**. The scale bar represents 50 µm

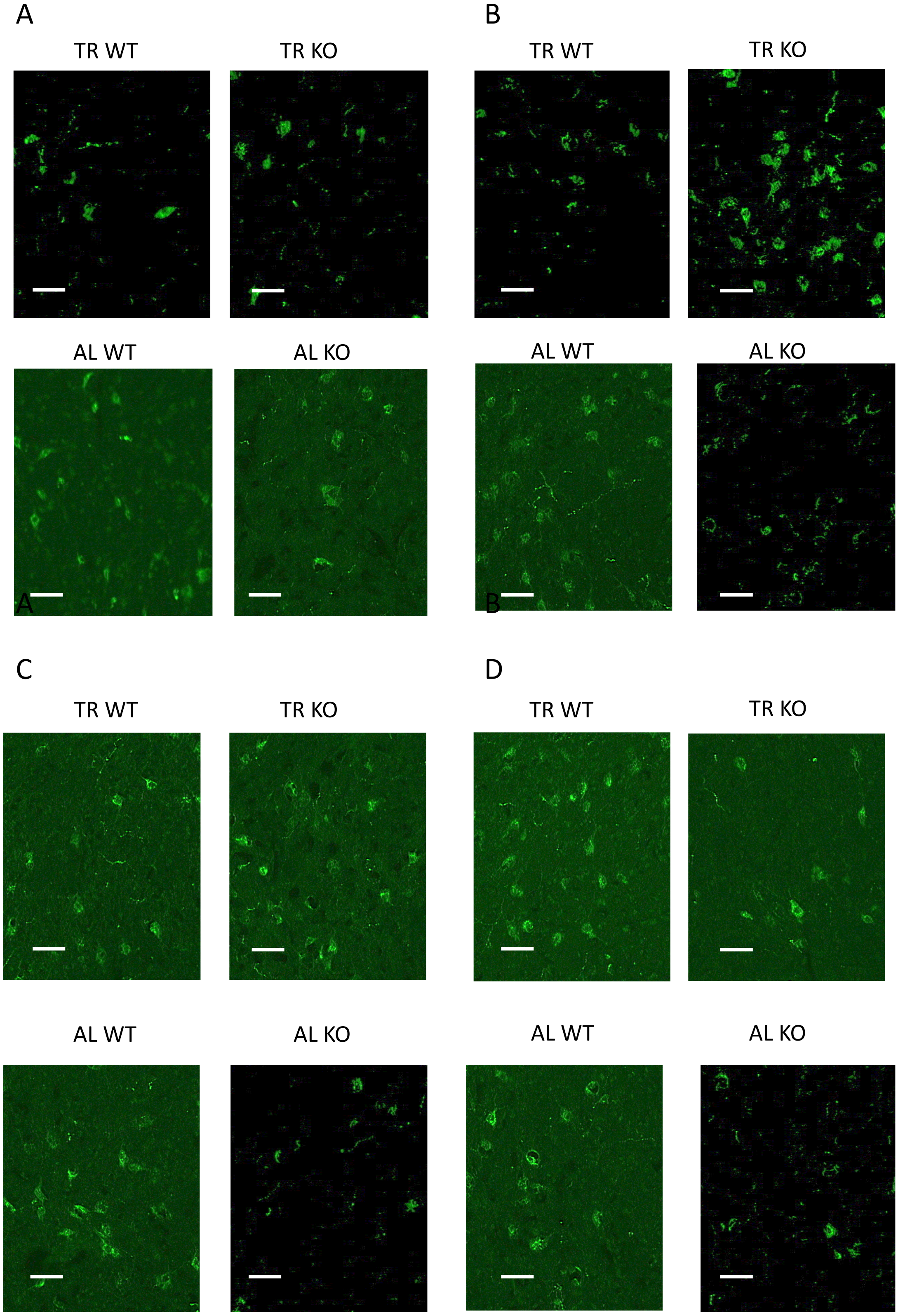

Supplement: Supplementary file 1 — Supplementary dataset 1. [file 41598_2020_60518_MOESM1_ESM.pdf]
